# Effects of 8-weeks of supplementation with a plant protein blend + creatine monohydrate on changes in maximal strength in resistance-trained males and females

**DOI:** 10.1080/15502783.2025.2550212

**Published:** 2025-08-25

**Authors:** Anthony M. Hagele, Kevin F. Holley, Alex C. Schrautemeier, James L. Tice, Joshua Iannotti, Joesi M. Krieger, Connor J. Gaige, Wyatt B. McLaughlin, Ralf Jäger, Chad M. Kerksick

**Affiliations:** a Lindenwood University, Exercise and Performance Nutrition Laboratory, Saint Charles, MO, USA; b Increnovo, LLC, Department of Kinesiology, Whitefish Bay, WI, USA

**Keywords:** Protein source, Creatine, resistance training, strength

## Abstract

**Background:**

Protein supplementation supports strength training adaptations with questions still evident over which source of protein is superior. Creatine monohydrate is well established for its ability to support exercise training adaptations, but limited research has explored any synergies that may exist between plant proteins and creatine monohydrate. This study sought to compare the changes in maximal strength and repetitions completed among resistance-trained males and females consuming a plant-based protein blend containing creatine.

**Methods:**

Sixty resistance-trained males (*n* = 31; 26 years, 81.5 ± 8.8 kg, 179.2 ± 7.8 cm) and females (*n* = 29; 23 years, 66.3 ± 9.4 kg, 161.0 ± 12.4 cm) participated in the 8-week double-blind, placebo-controlled, randomized study. For entry, participants were required to have 12 months of resistance training experience and achieve relative strength thresholds. Participants were randomly assigned (*n* = 15 each group) to supplement with either 48 g of a plant protein blend + creatine (PPCr), 48 g of a plant protein blend (PP), 49 g of whey protein (WP), or 48 g of a carbohydrate placebo (CHO) for 8 weeks, while following a 4-day per week structured resistance training program. Strength was assessed using the hex bar deadlift 1RM (HBDL) and bench press 1RM (BP) at Week 0 and Week 8. Work capacity (WC) was assessed by having each participant complete three sets of standardized loading (70% 1RM × 10 repetitions) followed by two sets of maximal repetitions at 80% 1RM with 2 minutes of rest between each set. 4 × 3 mixed factorial ANOVA with repeated measures was used to evaluate differences. A p-value of 0.05 was used to establish statistical significance and trends were considered if p-values were between 0.05 and 0.10.

**Results:**

Consistent increases in HBDL (7.5–12.2%), BP (7.2–10.9%), and WC (14.1–26.9%) were observed in all groups after the 8-week program resulting in statistically significant main effects of time (*p* < 0.001) for all variables. Group × time interactions HBDL (*p* = 0.743), BP (*p* = 0.604), and WC (*p* = 0.726) were not considered to be statistically significant.

**Conclusion:**

All groups experienced favorable increases in maximal strength in HBDL and BP and repetitions completed using the leg press, with no major differences between supplementation groups. Further investigations involving longer protocol duration with larger sample sizes may be warranted to further determine the potential efficacy of PPCr supplementation.

